# From slides to insights: Harnessing deep learning for prognostic survival prediction in human colorectal cancer histology

**DOI:** 10.1515/biol-2022-0777

**Published:** 2023-12-13

**Authors:** Jyoti Verma, Archana Sandhu, Renu Popli, Rajeev Kumar, Vikas Khullar, Isha Kansal, Ashutosh Sharma, Kanwal Garg, Neeru Kashyap, Khursheed Aurangzeb

**Affiliations:** Chitkara University Institute of Engineering and Technology, Chitkara University, Punjab, India; Department of Computer Science and Engineering, Punjabi University, Patiala, India; Department of Informatics, School of Computer Science, University of Petroleum and Energy Studies, Dehradun 248007, Uttarakhand, India; Department of Computer Science and Applications, Kurukshetra University, Kurukshetra, 136119, Haryana, India; Department of ECE, M.M. Engineering College, Maharishi Markandeshwar (Deemed to be University), Mullana, Ambala, Ambala, Haryana 134007, India; Department of Computer Engineering, College of Computer and Information Sciences, King Saud University, P.O. Box 51178, Riyadh 11543, Saudi Arabia; MM Institute of Computer Technology and Business Management Maharishi Markandeshwar (Deemed to be University) Mullana-Ambala, Haryana, 134007, India

**Keywords:** prognostic survival prediction, colorectal cancer, deep learning, histopathological analysis, retrospective multicenter study, image patches, hematoxylin & eosin staining

## Abstract

Prognostic survival prediction in colorectal cancer (CRC) plays a crucial role in guiding treatment decisions and improving patient outcomes. In this research, we explore the application of deep learning techniques to predict survival outcomes based on histopathological images of human colorectal cancer. We present a retrospective multicenter study utilizing a dataset of 100,000 nonoverlapping image patches from hematoxylin & eosin-stained histological images of CRC and normal tissue. The dataset includes diverse tissue classes such as adipose, background, debris, lymphocytes, mucus, smooth muscle, normal colon mucosa, cancer-associated stroma, and colorectal adenocarcinoma epithelium. To perform survival prediction, we employ various deep learning architectures, including convolutional neural network, DenseNet201, InceptionResNetV2, VGG16, VGG19, and Xception. These architectures are trained on the dataset using a multicenter retrospective analysis approach. Extensive preprocessing steps are undertaken, including image normalization using Macenko’s method and data augmentation techniques, to optimize model performance. The experimental findings reveal promising results, demonstrating the effectiveness of deep learning models in prognostic survival prediction. Our models achieve high accuracy, precision, recall, and validation metrics, showcasing their ability to capture relevant histological patterns associated with prognosis. Visualization techniques are employed to interpret the models’ decision-making process, highlighting important features and regions contributing to survival predictions. The implications of this research are manifold. The accurate prediction of survival outcomes in CRC can aid in personalized medicine and clinical decision-making, facilitating tailored treatment plans for individual patients. The identification of important histological features and biomarkers provides valuable insights into disease mechanisms and may lead to the discovery of novel prognostic indicators. The transparency and explainability of the models enhance trust and acceptance, fostering their integration into clinical practice. Research demonstrates the potential of deep learning models for prognostic survival prediction in human colorectal cancer histology. The findings contribute to the understanding of disease progression and offer practical applications in personalized medicine. By harnessing the power of deep learning and histopathological analysis, we pave the way for improved patient care, clinical decision support, and advancements in prognostic prediction in CRC.

## Graphical abstract

**Figure:**
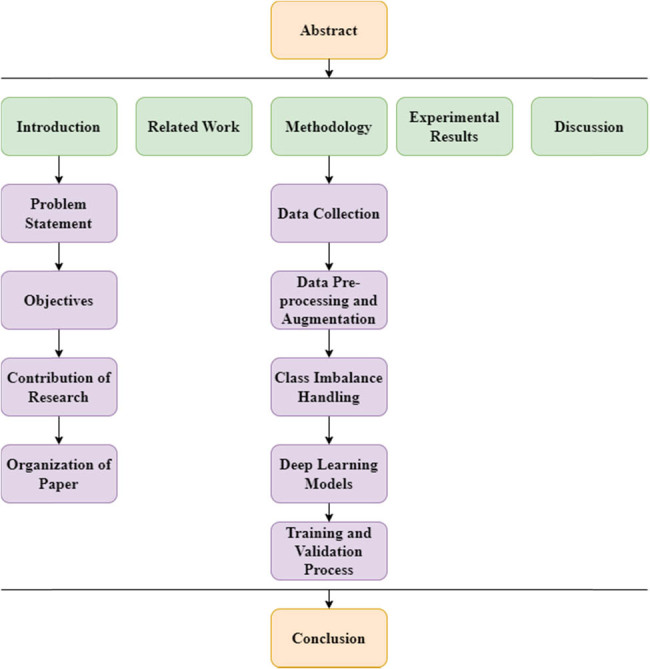

## 1 Introduction

Colorectal cancer (CRC) is a serious global health issue with a substantial mortality rate. The histopathological examination of tissue slides is crucial in assessing prognosis and developing treatment plans for patients diagnosed with CRC. Manually interpreting histology slides is marked by significant labor and is susceptible to variations in observations across different observers. To tackle these issues, methods based on deep learning have demonstrated considerable potential in automating the processing of histological images for prognostic survival prediction. CRC, or colon cancer or cancer of the rectal area, is a malignancy that specifically targets the colon or rectum, constituting integral components of the large intestinal tract. Typically, the progression commences with the emergence of nonmalignant formations known as polyps, which may evolve into malignant neoplasms. CRC is prevalent globally and contributes substantially to mortality rates associated with cancer [1].

The significance of CRC is its capacity to proliferate and metastasize without timely detection and intervention. When detected at an early stage, CRC frequently exhibits a high degree of treatability, leading to a notable enhancement in survival rates. Nevertheless, in the event of cancer advancement and metastasis, the therapeutic interventions become increasingly challenging, leading to a deterioration in the prognosis. Current colorectal cancer research focuses on enhancing early detection methods, devising more efficacious treatment approaches, and improving patient outcomes. Scientists are currently pursuing enhancing the precision and nonintrusiveness of screening modalities for the timely identification of CRC. These efforts include exploring blood tests, stool-based testing, and imaging techniques [2]. Investigating genetic mutations and changes linked to CRC can facilitate the identification of individuals with an elevated susceptibility to the disease and enable the customization of treatment strategies. Precision medicine is a paradigm that entails customizing treatment strategies by considering the unique attributes of an individual patient’s tumor. The aforementioned process encompasses targeted treatments, immunotherapies, and various personalized therapy techniques. Artificial intelligence and machine learning methodologies are currently being utilized to analyze extensive collections of information about patients, medical images, and genomic data to enhance the accuracy of diagnosis, treatment planning, and prognostication of treatment outcomes. Immunotherapy has demonstrated encouraging results in treating several forms of cancer, such as CRC. Current research efforts are investigating the utilization of immunotherapeutic drugs in conjunction with other treatment modalities to enhance the results of individuals diagnosed with CRC. The progression of surgical methodologies, including laparoscopic and robotic-assisted procedures, has resulted in less invasiveness, accelerated recovery, and enhanced results among individuals diagnosed with CRC. The primary objective of the research is to improve the overall well-being of individuals who have survived colorectal cancer utilizing survivorship programs, provision of psychosocial support, and effective management of treatment-related adverse effects [3].

### 1.1 Background

**Figure 1:**
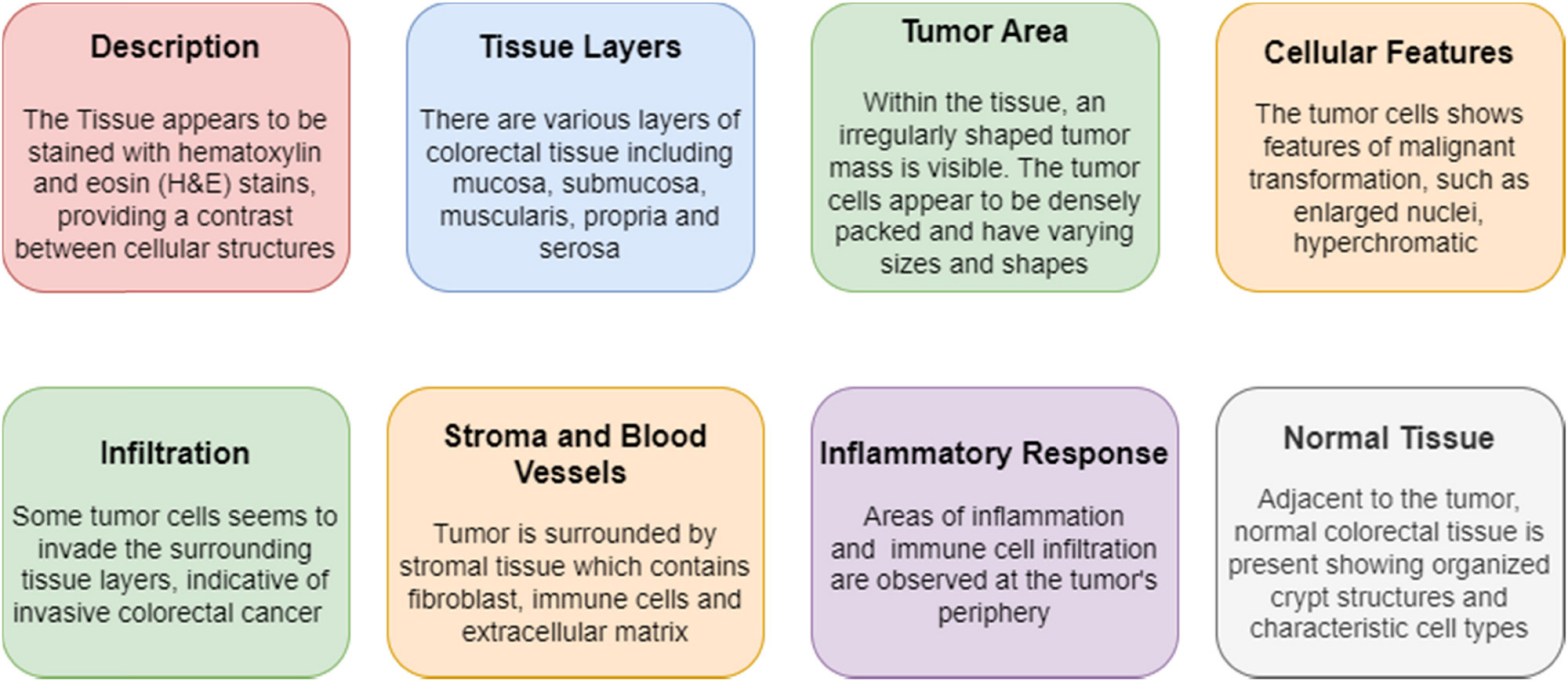
Typical human colorectal cancer histology slide 1.1 Background and Motivation.

A histology slide depicting human colorectal cancer reveals a delicate cross-section of colorectal tissue stained with hematoxylin and eosin (H&E) stains, which accentuate cellular structures. The slide showcases distinct tissue layers, encompassing the mucosa, submucosa, muscularis propria, and serosa. Notably, an irregular tumor mass dominates a portion of the tissue, its densely packed cells exhibiting varying sizes and shapes. Evident features of malignancy, such as enlarged nuclei, heightened nuclear staining (hyperchromasia), and conspicuous nucleoli, characterize these tumor cells. In addition, some cells infiltrate the surrounding tissue layers, signifying invasive colorectal cancer. The tumor’s microenvironment includes stromal tissue containing fibroblasts, immune cells, and an extracellular matrix, and blood vessels within the stroma suggest tumor’s capability to induce angiogenesis. Inflammatory responses and immune cell infiltration appear at the tumor’s edges, juxtaposed with adjacent normal colorectal tissue displaying well-structured crypt formations and characteristic cell types. This portrayal provides a general overview of the histological features that one might encounter in a human colorectal cancer histology slide, while recognizing that actual slides can vary depending on cancer stage, grade, and specific characteristics. Figure 1 presents working of histology slide of human colorectal cancer that typically consists of stained tissue sections under a microscope. The histology slide offers a complete visual representation of colorectal tissue, showcasing various layers that have been accentuated by applying hematoxylin and eosin staining. A tumor mass that exhibits uneven shape and is characterized by tightly packed cells of varying types indicates malignancy, suggesting cellular change. The presence of invasion into adjacent tissue layers indicates the presence of invasive colorectal cancer. The stromal tissue encompassing the tumor is composed of fibroblasts, immune cells, and discernible blood vessels, hence suggesting the presence of angiogenic capabilities. The presence of inflammatory regions and the infiltration of immune cells in the periphery of the tumor indicates the persistence of an immune response. The evaluation of tumor invasion can be effectively conducted by comparing it to adjacent normal colorectal tissue that exhibits well-organized crypt structures. The comprehensive histological examination described herein serves as a valuable tool for identifying cancer, facilitates the determination of its stage, and provides guidance for treatment strategies. Moreover, it underscores the significance of invasion, angiogenesis, and inflammation in comprehending the behavior of tumors and their potential to metastasize.

CRC is a prominent contributor to mortality rates associated with cancer globally. Precisely evaluating prognostic factors is crucial to ascertain suitable therapeutic approaches and enhance patient outcomes. Historically, the prognosis of CRC has depended on the human interpretation of histological slides, a process characterized by time consumption, subjectivity, and susceptibility to variability among different observers. To overcome these constraints, there is an increasing inclination toward utilizing deep learning methodologies to automate the examination of histology images and forecast survival outcomes in patients with CRC. Deep learning has exhibited exceptional achievements in diverse domains, encompassing medical picture analysis [4]. Convolutional neural networks (CNNs) and other deep learning architectures have demonstrated remarkable efficacy in several tasks, including but not limited to image classification, segmentation, and object recognition [5]. These models can acquire and extract complex information from images autonomously, thereby possibly identifying nuanced patterns and irregularities that could impact cancer prognosis [6].

**Figure 2:**
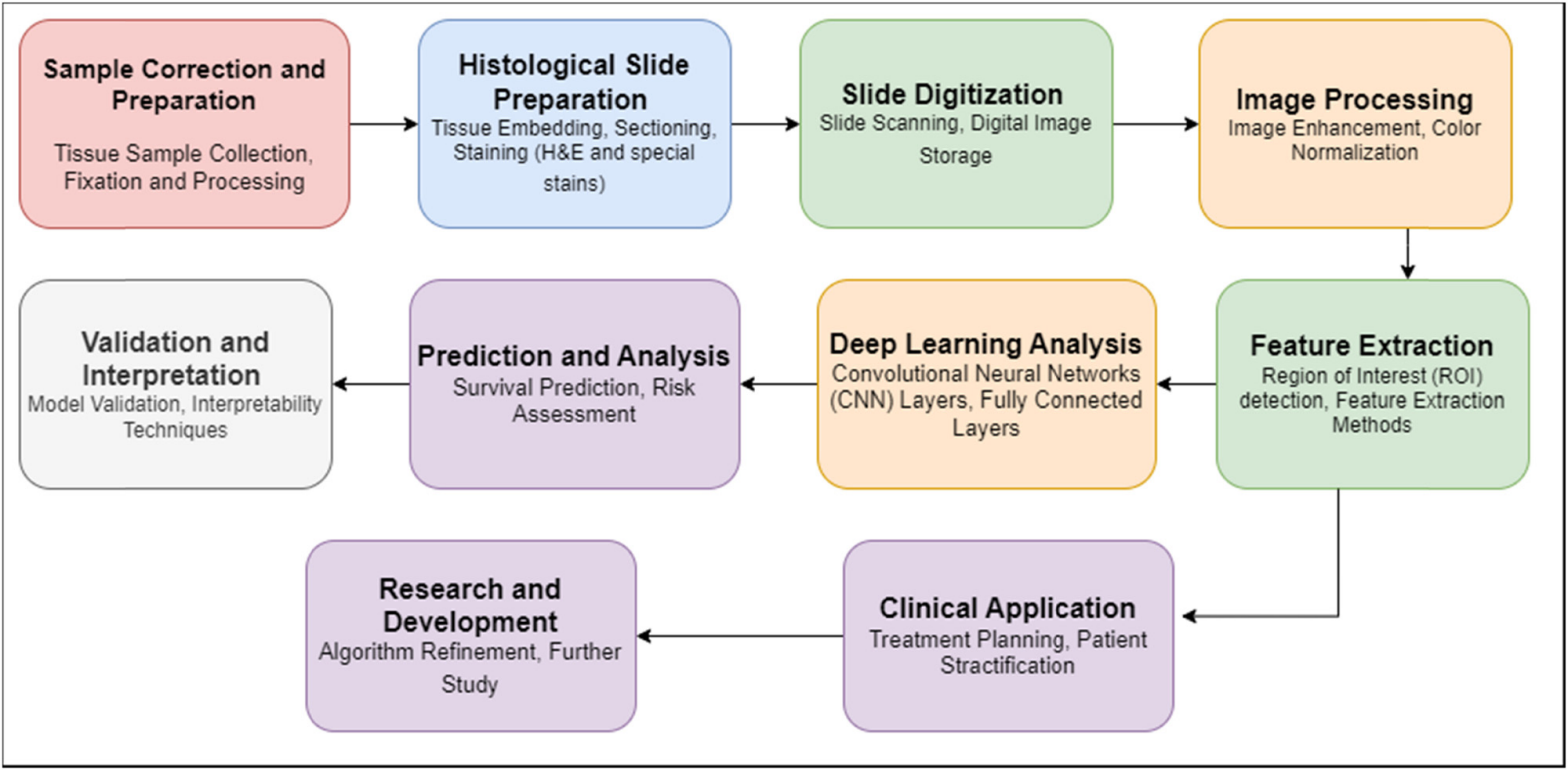
Block diagram of the essential stages within the domain of human colorectal cancer histology.

The block diagram in Figure 2 offers a concise visualization of the essential stages within the domain of human colorectal cancer histology. It systematically delineates the workflow from sample collection and preparation, encompassing tissue sample collection and fixation, to the subsequent histological slide preparation, involving tissue embedding, sectioning, and staining with H&E and specialized stains. The transformation of physical slides into digital format is illustrated through slide scanning and digital image storage blocks, leading to image preprocessing steps like enhancement and color normalization. Notably, feature extraction methods, including the detection of regions of interest and feature extraction itself, constitute pivotal aspects of the process. Deep learning techniques are harnessed for analysis, encompassing CNN layers and fully connected layers, culminating in predictions and risk assessments related to survival outcomes. Validation and interpretation phases comprise model validation and employ interpretability techniques. The clinical sphere is represented by blocks involving treatment planning and patient stratification, further highlighting the practical implications. The iterative nature of research and development is acknowledged through blocks for algorithm refinement and future study, eventually culminating in research findings and potential clinical impacts. While this representation is an abstraction, it effectively captures the fundamental stages of harnessing deep learning for prognostic survival prediction in human colorectal cancer histology.

The impetus for this study arises from the necessity for precise and effective prognostic evaluation in CRC utilizing histopathology slides. Using deep learning techniques makes it feasible to create automated systems that aid pathologists in examining and interpreting histology slides. This advancement could enhance the accuracy and dependability of prognostic predictions by ensuring greater consistency in the analysis process. Ultimately, this can facilitate the process of determining treatment decisions and improve the quality of patient care. Extensive datasets comprising histological pictures of CRC and healthy tissue have facilitated the development of powerful deep-learning models through training. The availability of these datasets facilitates the investigation of various deep-learning systems and algorithms to determine the optimal technique for predicting survival in CRC. Significant knowledge can be obtained by comparing and analyzing the performance of different models, hence shedding light on the predictive capacities of deep learning techniques in CRC histopathology. This study aims to provide a scholarly contribution to the area by assessing the effectiveness of several deep learning models using a comprehensive retrospective multicenter dataset [7]. The results can enhance the domain of histology-based estimation of survival in CRC, facilitating more precise and effective prognostic evaluation. The primary objective is to improve patient outcomes, optimize treatment approaches, and offer practitioners a valuable resource for personalized medicine in CRC.

### 1.2 Problem statement

The prognostic evaluation of CRC is paramount in informing treatment strategies and enhancing patient outcomes. Manually interpreting histopathology slides, considered the most reliable method of predictive assessment, is characterized by its labor-intensive nature, subjectivity, and susceptibility to variations among different observers. Consequently, there exists a necessity for automated and dependable methodologies that can accurately forecast survival outcomes in CRC utilizing histology slides. Conventional machine learning methods have exhibited potential in this domain; nevertheless, they frequently depend on manually designed features and may not comprehensively capture the intricate and nuanced patterns inherent in histology pictures. In contrast, deep learning methodologies have exhibited exceptional aptitude in image analysis endeavors, encompassing the classification and segmentation of medical images. By utilizing deep learning techniques, it becomes feasible to construct precise and effective models capable of autonomously acquiring and extracting pertinent characteristics from histology slides to forecast survival outcomes. Multiple obstacles must be confronted when considering using deep learning algorithms for predictive forecasting of survival in the histology of human colorectal cancer.

It is imperative to acquire extensive and heterogeneous datasets comprising histological pictures that include a wide range of tissue types and disease states. This is essential to ensure the effectiveness of model training and its ability to generalize accurately. Furthermore, carefully selecting and optimizing deep learning architecture appropriate for predicting CRC survival is paramount for optimal performance. Moreover, it is imperative to investigate the comprehension of deep learning algorithms within histopathological research to understand the characteristics and trends that play a role in predicting survival outcomes. Hence, the primary focus of this study revolves around the appropriate utilization of deep learning methodologies for prognostic survival prediction in the context of human colorectal cancer histology. This study aims to examine and assess various deep learning models utilizing a substantial retrospective multicenter dataset comprising histology pictures. The primary objective of this work is to offer valuable insights into the comprehension of the models employed and investigate the fundamental elements and patterns that contribute to the accurate prediction of survival outcomes. The primary objective of this study is to solve the preceding to establish a connection between survival prediction based on histology and deep learning techniques. The final goal is to develop a beneficial tool that can enhance prognostic assessment in CRC and facilitate the implementation of personalized treatment plans.

### 1.3 Objectives

The primary objective of this research is to establish a connection between survival prediction based on histology and deep learning techniques. This study seeks to make significant advancements in the area, ultimately leading to improved patient outcomes and better knowledge about the treatment of CRC. The objective of this study is to investigate and evaluate the efficacy of several deep learning models, such as CNN, VGG16, VGG19, DenseNet201, InceptionResNetV2, and Xception, in predicting predictive survival outcomes in human colorectal cancer histology. This study aims to determine the optimal deep-learning models for correctly predicting survival outcomes using histology pictures. The study employs an extensive dataset of 100,000 distinct picture patches derived from histological images of human colorectal cancer and normal tissue stained with H&E. The aim is to utilize the given dataset to train and assess the deep learning models, guaranteeing strong performance and generalizability. This study aims to evaluate the efficacy of deep learning models by employing diverse metrics, including precision, recall, accuracy, and loss. This study aims to assess the quantitative effectiveness of the models for foreseeing survival outcomes and to compare their respective performances. This study aims to investigate the interpretability and visualization of deep learning models within the specific domain of colorectal cancer histology. This study seeks to understand the characteristics and trends that predict survival, improving our comprehension of the fundamental elements that impact prognosis. The primary aim of this study is to provide a valuable contribution toward enhancing prognostic evaluation in the context of human colorectal cancer. This study aims to utilize deep learning methodologies to create automated systems that can aid pathologists in the analysis and interpretation of histology slides. The primary aim is to offer precise and effective prognostic forecasts by examining histopathology data, facilitating clinical decision-making and the development of individualized treatment approaches.

### 1.4 Contribution of the research

The study offers substantial advancements in the area of histology-based prognostication of survival in colorectal cancer through the use of deep learning methodologies. It encompasses assessing various models, employing a dataset from multiple medical centers, investigating interpretability, and furnishing valuable insights for aiding clinical decision-making. The preceding contributions enhance the comprehension and implementation of deep learning techniques in prognostic evaluation, ultimately yielding advantages for patients and physicians in combating colorectal cancer.

- *Comparative analysis of deep learning architectures:* To predict predictive survival in colorectal cancer histology, this study undertook a thorough comparison investigation of different deep learning architectures, namely, DenseNet201, CNN, InceptionResNetV2, VGG16, VGG19, and Xception. This study facilitates the identification of the most productive models for this activity.
- *Visualization and interpretation techniques:* The study included visualization and interpretation tools, such as activation maps, feature visualization, and attention mechanisms, to augment the comprehension of the decision-making process of deep learning models. These methodologies offer valuable perspectives on the prominent characteristics and areas that impact prognostications of survival.
- *Practical applications in personalized medicine:* The deep learning models that have been constructed possess practical applications within the field of personalized healthcare since they demonstrate the ability to forecast survival outcomes for specific patients with colorectal cancer reliably. This can assist healthcare professionals in customizing treatment strategies and enhancing the quality of patient care.
- *Foundation for future research:* The present research establishes a fundamental basis for future investigations about prognostic survival prediction in colorectal cancer histology. The models, dataset, and preprocessing approaches that have been based on this study can be utilized by fellow researchers to extend the scope of this research, explore novel research inquiries, and construct sophisticated models and methodologies.

### 1.5 Organization of the article

This article is structured into six distinct sections. Section 1 offers crucial contextual information, encompassing the historical background, underlying motivation, and problem statement. In addition, it outlines the research objectives and emphasizes the substantial contribution that can be made by utilizing deep learning methods for prognostic survival prediction in the histology of human colorectal cancer. Section 2 provides an extensive examination of pertinent literature, including the prognostication of colorectal cancer, histological analysis, and the use of deep learning in processing medical images. A concise overview of prior research on survival prediction based on histology is presented. Section 3 presents a comprehensive account of the methodology, encompassing the elucidation of the dataset employed, the preprocessing procedures undertaken, the management of class imbalance, and an overview of the diverse deep learning architectures employed in the study, including the CNN, DenseNet201, InceptionResNetV2, VGG16, VGG19, and Xception. The experimental results of the study are described in Section 4, encompassing the performance metrics and assessment criteria employed to demonstrate and analyze the consequences of various topologies. Following this, Section 5 delves into a comprehensive discourse, examining the implications of the findings, recognizing constraints, and proposing prospective avenues for further research. Section 6 provides a summary of the scientific contributions and practical applications, particularly emphasizing the clinical implications of the study’s findings.

## 2 Related work

Numerous prior studies have been undertaken, which relate to investigating prediction of survival in colorectal cancer by deep learning techniques based on histology. Numerous previous experiments have been conducted to explore the predictive value of histology-based prediction of survival in diverse cancer types, including colorectal cancer. This research has investigated the utilization of various methodologies, encompassing conventional machine learning algorithms and deep learning techniques. The studies mentioned earlier exhibit an increasing inclination toward employing deep learning and intelligent technology methodologies to predict survival rates in colorectal cancer, using histology slides as the primary data source. The researchers offered significant contributions by presenting valuable perspectives on the approach, performance, and issues of deep learning-based approaches within this domain. The study conducted more exploration into deep learning models, employing a substantial dataset and offering novel contributions to the part of histology-based prognostication in colorectal cancer. The subject of medical imaging analysis has witnessed considerable interest in applying deep learning techniques. Numerous prior studies have examined using deep learning methodologies in diverse medical imaging endeavors.

In their study, Wang et al. successfully devised and verified a deep-learning algorithm to identify polyps during colonoscopy. Their findings highlight the promising role of machine learning in enhancing the precision of early diagnosis of colorectal cancer [8]. The study conducted by Sun et al. investigated the impact of TRIM29 on the inhibition of cancer stem cell-like properties in pancreatic ductal adenocarcinomas, providing valuable insights into future therapeutic strategies for this very aggressive form of cancer [9]. The study by Ren et al. examined the phenomenon of keratin 23 overexpression and its impact on facilitating the migration of ovarian cancer cells via the epithelial–mesenchymal transition process [10]. In their study, Pan et al. examined the significance of utilizing AGR2 and KRT5 as immunomarker pairings in differentiating squamous cell carcinoma of the lungs from other subtypes of lung cancer [11]. Dwyer Nield et al. conducted a study investigating the possible role of PPARγ agonism in impeding the advancement of premalignant lesions in a mouse squamous cell carcinoma of the lung model. Their findings provide valuable insights into future therapeutic approaches [12]. Huang et al. examined the involvement of TRIM group proteins in carcinogenesis, cancer formation, and treatment resistance, underscoring the significance of these amino acids in cancer biology [13]. Han et al. aimed to examine the transcriptional dysregulation of TRIM29 and its role in the development of colorectal cancer by affecting pyruvate kinase-mediated glucose metabolism [14]. Qiao et al. investigated the regulatory function of TRIM29 in the advancement of ovarian cancer, specifically focusing on its impact on the SETBP1/SET/PP2A axis. Their study yielded valuable insights into the underlying molecular mechanisms associated with this process [15]. The study by Hao et al. examined the impact of TRIM29 on the bioenergetics of cancer cells in the pancreas using miRNA and DDX3X recruitment. The findings shed light on the potential involvement of TRIM29 in the progression of pancreatic cancer [16]. The study conducted by Ray and Mukherjee provides a complete overview of the roles played by TRIM protein in breast cancer, focusing specifically on their altered expression and oncogenic effects in this disease [17].

The study conducted by Lei et al. investigated the ability of TRIM29 to counteract oxaliplatin tolerance in cells with colon cancer, hence offering novel approaches to address drug resistance [5]. Cockram et al. provided a comprehensive analysis of the involvement of ubiquitination in regulating inflammation-induced cell death and its implications in cancer. The research shed valuable insights into the complex molecular mechanisms behind these processes [18]. Razzaghdoust et al. explored the correlation between immunohistochemical markers and the response to chemotherapy with neoadjuvant therapy and overall survival in individuals with muscle-invasive bladder cancer. The authors underscored the significance of tailored treatment strategies in this context [19]. Chehade et al. conducted a study to investigate the application of medical imaging, explicitly employing a feature engineering method, to classify lung and colon cancer. The study emphasized the potential of imaging techniques in the field of diagnostics [20]. Kather et al. have published a dataset comprising histological pictures of both human colorectal cancer and normal tissue. This dataset is an excellent resource for researchers developing machine-learning systems for diagnosing colorectal cancer [21]. Tamang and Kim conducted a comprehensive examination of deep learning methodologies utilized in the context of colorectal cancer diagnosis. The authors thoroughly analyzed the diverse methods and algorithms employed within this domain [22]. Davri et al. conducted a comprehensive analysis centered on using deep learning techniques to examine histopathological pictures to diagnose colorectal cancer. Their study provided a concise overview of the latest developments in this field [23]. Islam et al. investigated the possible applications of natural products for preventing and treating colon and colorectal cancer. Their study highlighted the significance of adopting a comprehensive approach to managing cancer [24]. Collins et al. examined the application of machine learning and hyperspectral imaging for automated colon and esophagogastric cancer identification. The authors emphasized the promising prospects of employing modern imaging methodologies in cancer detection [25]. Sakr et al. introduced a highly effective deep-learning methodology for identifying colon cancer, thereby significantly contributing to advancing diagnostic tools based on artificial intelligence [26]. Alsanea et al. offered valuable insights into the occurrence, longevity, and demographic characteristics of colorectal cancer in Saudi Arabia. The findings underscore the necessity of developing customized national cancer care strategies that consider various locations’ unique needs and circumstances [27].

These earlier works provide valuable insights into the histopathological factors that influence prognosis and patient outcomes in colorectal cancer. They highlight the significance of various factors in predicting lymph node involvement, microsatellite instability, and overall survival. The research in the field of colorectal cancer prognosis and histopathological analysis builds upon these studies to develop advanced techniques, such as deep learning, for accurate and automated prognostic assessment. These earlier works demonstrate the wide-ranging applications of deep learning in medical image analysis. They highlight the potential of deep learning models, particularly CNNs, in various imaging tasks, including classification, segmentation, and detection. The research in this field continues to explore and refine deep learning techniques to improve accuracy, efficiency, and clinical utility in medical image analysis.

The present state of colorectal cancer diagnosis and histological analysis reveals various areas where further research is needed. The existing body of literature heavily depends on datasets that are constrained in scope, highlighting the need to get more extensive and diversified datasets that span a more comprehensive range of cancer stages, tissue types, and demographic variables. Integrating multi-omics data and conducting thorough clinical validation in real-world situations is crucial for augmenting the comprehensiveness and applicability of artificial intelligence models. The issues of interoperability with electronic health records, explainability of AI predictions, and resolving imbalanced datasets, especially in rare events, continue to be of utmost importance. Using real-time processing in endoscopy and integrating clinical and histopathological data presents exciting opportunities for enhancing clinical practice. Furthermore, examining ethical and regulatory factors thoroughly while implementing artificial intelligence to guarantee responsible and secure deployment within the healthcare sector is imperative. Resolving these deficiencies will propel the discipline forward, yielding advantages for healthcare practitioners and individuals seeking medical treatment.

## 3 Methodology

This section provides an overview of the steps followed in the research to address the research objectives. It describes the data acquisition, preprocessing, deep learning model selection and training, performance evaluation, and interpretability analysis. The methodology serves as a roadmap for conducting the study and forms the foundation for the subsequent analysis and discussion.

**Figure 3:**
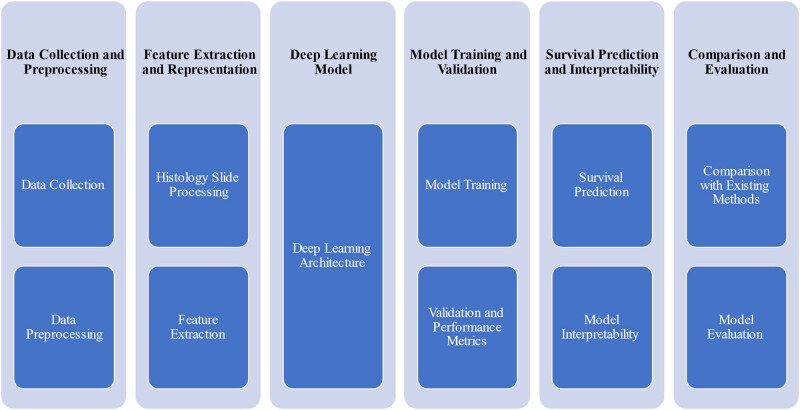
Overview of the steps followed in the research to address the research objectives.

As shown in Figure 3, the research embodies an extensive and multifaceted inquiry into the utilization of cutting-edge deep learning methodologies to prognosticate survival outcomes among individuals afflicted by colorectal cancer. The research framework is intricately structured to encompass a comprehensive trajectory, commencing with a foundational introduction that establishes the gravity of colorectal cancer within the medical landscape and underscores the pivotal role of prognostic survival prediction in tailoring personalized therapeutic interventions. Central to the research is the nuanced exploration of histology slides, elucidating their integral contribution to the diagnosis and prognostic stratification of cancer cases. This journey extends into a comprehensive literature review, encompassing a meticulous assessment of prevailing methodologies while underscoring the transformative potential of deep learning paradigms in the realm of medical image analysis and survival prognosis. The crux of the research endeavor unfolds through meticulous data collection and preprocessing, where the acquisition and meticulous curation of CRC histology slides form the bedrock upon which subsequent analysis is constructed [28]. The underpinning deep learning architecture emerges as a pivotal element, entailing a detailed exposition of chosen models and components, including the intricate mechanics of CNNs and attention mechanisms, which imbue the research with predictive potency. The intricate process of feature extraction and representation is navigated, traversing the complex terrain of translating histological intricacies into informative inputs for the model. Critical to the research progression is the iterative cycle of model training and validation, elucidating strategies employed to enhance performance and robustly assess the veracity of predictions. Subsequent stages delve into the application of trained models for survival prognostication, accompanied by insightful exploration into their interpretability, bridging computational predictions with the nuances of clinical decision-making [29]. Ultimately, the research culminates in a comprehensive discussion segment that interweaves empirical findings with their far-reaching clinical implications. This discourse poignantly accentuates the potential paradigm-shifting impact of the proposed approach on patient-centric care and treatment modalities. In essence, this research venture traverses uncharted territories at the intersection of deep learning prowess and colorectal cancer histology, poised to unravel insights that hold the potential to reshape the landscape of prognostic survival prediction, engendering a transformative shift in our understanding and management of this complex malignancy.

The overarching framework for research into the prognostication of survival in colorectal cancer using deep learning may be delineated as follows:

- *Data acquisition and collection:* The data are acquired from reliable sources, including the NCT Biobank and UMM pathology archive. The collection comprises formalin-fixed paraffin-embedded (FFPE) tissue samples about colorectal cancer, encompassing initial tumor slides, liver metastases, and nontumorous regions. Comprehensively portraying histological pictures, including a varied range of tissues and disease states, is imperative.
- *Data preprocessing and augmentation:* The histological pictures gathered undergo several preprocessing procedures. These procedures involve color normalization, scaling the images to a standardized size (224 × 224 pixels), cleaning the data to eliminate extraneous artifacts, and using image-enhancing techniques, including contrast modification and noise reduction. Data augmentation strategies are utilized to enhance the diversity of a dataset. These techniques include random rotation, flipping, scaling, cropping, adjusting brightness and contrast, and applying elastic transformations.
- *Class imbalance handling:* Various techniques are utilized to mitigate the issue of class imbalance. Oversampling techniques aim to enhance the presence of underrepresented minority classes within a dataset. Undersampling courses aim to decrease the representation of the majority class. During the training of a model, the technique of class weighting assigns greater significance to the minority classes. Hybrid methodologies have the potential to integrate oversampling and undersampling approaches.
- *Deep learning model selection:* Different deep learning algorithms are selected depending on their efficacy in tasks related to the processing of images. CNNs are a class of deep learning models widely used in computer vision tasks. Some popular CNN architectures include DenseNet201, InceptionResNetV2, VGG16, VGG19, and Xception. These architectures have been developed and optimized for various image classification and recognition tasks. These models possess unique architectural attributes and advantages.
- *Model training and validation:* The dataset is partitioned into two subsets: the training and validation sets. Data preparation is crucial in data analysis since it involves applying several techniques to standardize and improve the dataset. The deep learning model that has been chosen is initialized, and the hyperparameters are adjusted. The training process consists of several epochs, wherein the model iteratively reduces the loss function. The model’s performance is evaluated periodically on the validation set, which serves as a basis for making necessary improvements to the model.
- *Performance evaluation:* Various performance metrics are calculated to evaluate a model’s performance, including accuracy, recall, F1 score, precision, AUC-ROC, and loss. The findings are displayed in the tabular format and visually represented through graphical charts. These criteria facilitate the assessment of the efficacy of various models in predicting survival results.

### 3.1 Data collection and acquisition

The data collection and acquisition process in the research involved obtaining a large retrospective multicenter dataset of histological images from reputable sources. The researchers identified reputable sources from which to collect the histological images. In this case, the dataset was sourced from the NCT Biobank (National Center for Tumor Diseases, Heidelberg, Germany) and the UMM pathology archive (University Medical Center Mannheim, Mannheim, Germany). These biobanks and archives are known for their collection and storage of FFPE tissue samples for research purposes [21]. The researchers identified the specific tissue samples to be included in the dataset. The samples were selected based on their relevance to colorectal cancer, including CRC primary tumor slides, tumor tissue from CRC liver metastases, and nontumorous regions from gastrectomy specimens. This selection aimed to ensure a diverse representation of histological images encompassing different tissue types and disease states. The selected tissue samples were manually processed to extract histological images. This likely involved retrieving the FFPE tissue blocks and sectioning them to obtain thin slices suitable for histological analysis. The slides were then stained using H&E staining, a commonly used staining method that highlights cellular structures and facilitates histopathological examination. The histological slides were digitized to convert them into high-resolution digital images. Digital scanning systems or whole-slide imaging scanners were used to capture the images, preserving the fine details and cellular morphology present in the original slides. This digitization process allowed for easy storage, sharing, and analysis of the histological images. From the digitized histological images, a dataset was created by extracting nonoverlapping image patches. These patches were likely selected based on predefined criteria, such as size (224 × 224 pixels) and representation of different tissue classes (e.g., adipose, debris, lymphocytes, normal colon mucosa). The dataset was carefully curated to ensure it captured the necessary variability and provided an adequate representation of the histological characteristics of colorectal cancer and healthy tissue.

### 3.2 Data preprocessing and augmentation

Data preprocessing and augmentation are crucial steps in preparing the dataset for analysis and improving the performance and generalization of deep learning models. Color normalization is often performed to address staining variations across histological images. It ensures consistent color representation by removing the effects of staining inconsistencies. One commonly used method is Macenko’s method, which normalizes the color distribution across the images. Rescaling the images to a uniform size is necessary to ensure compatibility and efficient processing. In the case of the research, the histological images were likely rescaled to a standardized size, such as 224 × 224 pixels, which is a common input size for deep learning models. Data cleaning involves removing any irrelevant or noisy artifacts from the images. It may include removing image borders, text, or other artifacts that do not contribute to the analysis or may introduce biases. Image enhancement techniques can be applied to improve image quality and highlight relevant features. This may involve contrast adjustment, noise reduction, or sharpening to enhance the visibility of structures and patterns in the histological images. Data augmentation techniques are employed to increase the diversity and variability of the dataset, which helps improve the model’s ability to generalize and handle different variations in the input data. The images can be randomly rotated by a certain degree to introduce variations in orientation. This helps the model become invariant to the rotation of the histological features. Horizontal or vertical flipping of the images can be performed to introduce mirror images. This allows the model to learn from different perspectives and helps improve robustness. Images can be scaled to different sizes or randomly cropped to extract smaller regions of interest. This introduces variability in the image size and focus, enabling the model to learn from different spatial contexts. Modifying the brightness and contrast of the images can be used to simulate different lighting conditions. This augments the dataset with variations in illumination, making the model more robust to lighting differences. Elastic transformations deform the images nonrigidly, mimicking tissue distortions or morphological variations. This technique can capture tissue deformation patterns and improve the model’s ability to handle variations in tissue structure. Adding random noise to the images can help the model learn to be more robust to noise or artifacts present in real-world data. By performing data preprocessing and augmentation, the research aims to standardize the data, enhance image quality, increase dataset diversity, and improve the model’s ability to generalize and make accurate predictions. These steps are crucial for achieving reliable and robust performance in histology-based survival prediction using deep learning [29].

### 3.3 Class imbalance handling

Class imbalance handling is an essential step in addressing the disproportionate distribution of samples across different classes in a dataset. Class imbalance occurs when the number of instances in one class significantly outweighs the number of instances in another class. In the context of histology-based survival prediction research, class imbalance handling techniques are employed to mitigate the potential bias introduced by imbalanced class distributions. Oversampling techniques aim to increase the representation of minority classes by generating synthetic samples. This can be achieved through methods such as random duplication or by using more advanced techniques like synthetic minority oversampling technique, which synthesizes new samples by interpolating between existing instances of the minority class. Undersampling techniques involve reducing the number of instances in the majority class to achieve a more balanced distribution. Random undersampling randomly removes instances from the majority class until a desired balance is reached. However, undersampling can result in the loss of potentially useful information from the majority class.

Class weighting assigns different weights to different classes during model training to give higher importance to the minority class. This allows the model to focus more on correctly classifying the minority class instances, effectively reducing the impact of class imbalance. The weights can be used during loss calculation, where the contribution of each class to the overall loss is weighted based on its imbalance ratio. Hybrid approaches combine oversampling and undersampling techniques to address class imbalance. These methods aim to strike a balance between increasing the representation of minority classes while maintaining a manageable dataset size. For example, researchers may oversample the minority class and then apply undersampling to the majority class to achieve a more balanced dataset. The choice of class imbalance handling technique depends on several factors, including the severity of class imbalance, the size of the dataset, and the specific requirements of the research. It is crucial to carefully select and evaluate the appropriate technique to prevent overfitting, information loss, or the introduction of new biases. The class imbalance handling should be performed during the training phase and not during the evaluation or testing phase to ensure fair and unbiased performance evaluation. The goal is to provide the model with a balanced representation of different classes, allowing it to learn from all classes equally and make accurate predictions for all classes, including the minority class.

### 3.4 Deep learning models

The deep learning models were chosen for their effectiveness in image analysis tasks, including histopathological analysis. Each model has its own architectural characteristics and strengths, making them suitable for different types of data and research objectives. By using a combination of these models, the research aimed to explore their performance and identify the most effective model for prognostic survival prediction in colorectal cancer histology.

CNN is a widely used deep learning architecture for image analysis tasks. It consists of multiple convolutional layers followed by pooling layers and fully connected layers. CNNs are known for their ability to extract hierarchical features from images and have been successfully applied to various computer vision tasks. DenseNet is a type of CNN architecture that introduces direct connections between layers, allowing for more efficient information flow and gradient propagation. DenseNet201 is a specific variant of DenseNet with 201 layers. It has shown strong performance in image classification and has been widely used in medical image analysis tasks. InceptionResNetV2 is a deep learning architecture that combines the Inception module and residual connections. It uses multiple parallel convolutional layers of different sizes to capture various scales of image features. InceptionResNetV2 has demonstrated excellent performance in image recognition tasks and is known for its high accuracy. Visual geometry group (VGG) models are deep CNNs with either 16 or 19 layers. They have a simple and uniform architecture, with small convolutional filters and max pooling layers. VGG models are known for their excellent performance on image classification tasks and have been widely used as benchmark models. Xception is a deep learning model inspired by the Inception architecture. It employs depthwise separable convolutions, which separate spatial and channel-wise convolutions, reducing computational complexity while maintaining performance. Xception has shown strong performance on image classification tasks and offers a good balance between accuracy and efficiency [21].

### 3.5 Training and validation procedures

The training and validation procedures are crucial steps in the research process for training and evaluating deep learning models for prognostic survival prediction in human colorectal cancer histology. A training set is created by randomly selecting a portion of the available dataset. The training set consists of histological images of colorectal cancer and corresponding labels or annotations indicating the survival outcomes. The size of the training set may vary depending on the dataset and the complexity of the task [22]. A validation set is created by partitioning a portion of the dataset separate from the training set. The validation set is used to monitor the model’s performance during training and make decisions regarding hyperparameter tuning, model selection, and potential overfitting. The validation set should be representative of the underlying distribution of the data and include samples from all classes. Before training, the histological images undergo preprocessing steps to standardize and enhance the data. This may include resizing the images to a consistent resolution, normalizing pixel values, applying data augmentation techniques to increase dataset diversity, and potentially handling class imbalance if present. The chosen deep learning model (e.g., CNN, DenseNet201, InceptionResNetV2, VGG16, VGG19, Xception) is initialized with appropriate weights [23]. Depending on the availability of pretrained models, the weights can be initialized with random values or transferred from pretrained models trained on large-scale datasets (e.g., ImageNet) to leverage learned features from learning models and optimizing its parameters. The model is trained using gradient-based optimization algorithms such as stochastic gradient descent or Adam. The loss function, which measures the discrepancy between predicted and actual survival outcomes, is minimized during training. The training process typically consists of multiple iterations called epochs, where the entire training set is processed by the model. Hyperparameters, such as learning rate, batch size, regularization techniques, and optimizer parameters, may significantly impact the model’s performance. These hyperparameters are tuned during the training process, often using techniques like grid search, random search, or more advanced optimization algorithms, to find the optimal combination of hyperparameters that yields the best performance on the validation set. Throughout the training process, the model’s performance is periodically evaluated using the validation set. Various evaluation metrics, such as accuracy, precision, recall, and loss, are computed to assess the model’s performance and identify potential overfitting or underfitting issues. The model’s performance on the validation set helps in guiding the model selection process and determining when to stop training. Based on the validation set performance, adjustments can be made to the model architecture, hyperparameters, or preprocessing techniques to improve the model’s performance further. This iterative refinement process involves repeating the training, validation, and evaluation steps until a satisfactory model is obtained [24]. Table 1 presents a tabular representation outlining the proof of concept (PoC) about the utilization of deep learning techniques for predicting survival outcomes in colorectal cancer histology.

**Table 1:** PoC about the utilization of DL for predicting survival outcomes in CRC histology

PoC Scenario	Description	Functioning	Operation	Outcome
Retrospective clinical data	Collected historical clinical data, including histological images and survival outcomes	Applied the deep learning framework to this dataset	Analyzed the model’s predictions vs actual outcomes	Demonstrated the framework’s ability to accurately predict survival outcomes using historical patient data
Prospective clinical trial	Collaborated with a clinical trial, integrated framework, predicted patient survival outcomes as they enrolled	Integrated deep learning into the trial’s workflow	Monitored the framework’s impact on treatment decisions	Assessed whether the framework informed personalized treatment plans during a real clinical trial, potentially improving patient survival
Multicenter data validation	Gathered histological data from multiple healthcare centers	Applied the deep learning framework to this multi-center data	Evaluated performance across diverse centers	Validated the framework’s robustness and generalizability in real-world healthcare settings with varying data characteristics
Clinical decision support	Integrated your framework as clinical decision support	Clinicians used framework predictions for patient care	Monitored alignment with clinical decisions	Evaluated the framework’s value in assisting clinicians to provide personalized treatment plans, potentially improving patient outcomes
Patient outcomes monitoring	Implemented your framework for monitoring survivor outcomes	Predicted long-term survival based on follow-up histological assessments	Continuously tracked patient outcomes	Assessed the framework’s ability to provide accurate long-term survival predictions, aiding in the development of effective survivorship care plans
Telemedicine and remote consultation	Deployed framework for remote consultations in underserved areas	Patients submitted histological images for analysis	Monitored patient outcomes and assessed the framework’s impact	Determined if the framework enhanced healthcare access and improved patient outcomes in remote or resource-limited settings

## 4 Experimental results

The performance metrics and evaluation criteria enable the assessment of the deep learning models’ predictive capabilities and the comparison of different models’ performance. Researchers often analyze and report these metrics to demonstrate the effectiveness of their proposed models and to evaluate their suitability for prognostic survival prediction in CRC histology. Accuracy measures the proportion of correctly predicted survival outcomes compared to the total number of predictions. It provides an overall assessment of the model’s correctness in predicting survival. Precision calculates the proportion of true positive predictions (correctly predicted positive outcomes) to the total number of positive predictions (both true positives and false positives). It indicates the model’s ability to correctly identify positive cases. Recall, also known as sensitivity or true positive rate, measures the proportion of true positive predictions to the total number of actual positive cases. It evaluates the model’s ability to capture all positive cases. The F1 score is the harmonic mean of precision and recall. It provides a balanced measure of the model’s precision and recall performance, considering both false positives and false negatives. AUC-ROC evaluates the model’s performance in binary classification tasks by measuring the trade-off between true positive rate (sensitivity) and false positive rate. It provides a single value that summarizes the model’s discriminatory power across different classification thresholds. The loss function represents the discrepancy between the predicted survival outcomes and the actual survival outcomes. Different loss functions can be used based on the specific task, such as binary cross-entropy loss or mean squared error loss. The validation loss measures the performance of the model on the validation set. It provides an indication of the model’s generalization capability and helps identify potential overfitting or underfitting issues.

The performance measures of different DL architectures in the research demonstrate significant results. DenseNet201 and Xception have been identified as the leading models, with outstanding accuracy of 99.95%, highlighting their predictive skills. The DenseNet201 model achieved a precision value of 100%, indicating its high accuracy in properly recognizing positive cases. Similarly, the InceptionResNetV2 and Xception models reached almost 100% precision levels, further highlighting their skill in accurately detecting positive situations. The high recall values, notably the 99.925% achieved by Xception, demonstrate its ability to identify a significant portion of true positives effectively. Although CNN and VGG19 models had marginally poorer outcomes in specific criteria, they exhibited outstanding performance. DenseNet201 and Xception demonstrated exceptional performance in terms of validation accuracy, hence emphasizing their considerable potential for clinical applications. The results in Table 2 indicate the efficacy of deep learning models in prognostic survival prediction for CRC histology.

**Table 2:** Results of Normal Result DNN

	CNN	DenseNet201	InceptionResNetV2	VGG16	VGG19	Xception
Accuracy	70.525	99.95	99.85	99.8	99.225	99.95
Validation accuracy	70.6	94.8	92.8	93.8	90.8	95.4
Precision	75.11835	100	99.8998	99.79995	99.34853	99.94999
Validation precision	100	95.15151	93.34677	93.95161	91.27789	95.55556
Recall	63.725	99.875	99.7	99.775	99.125	99.925
Validation recall	63.6	94.6	92.6	93.2	90	94.6
Loss	0.696141	0.098516	0.111037	0.112601	0.151397	0.090078
Validation loss	0.729109	0.283531	0.386633	0.377719	0.485996	0.285021

**Figure 4:**
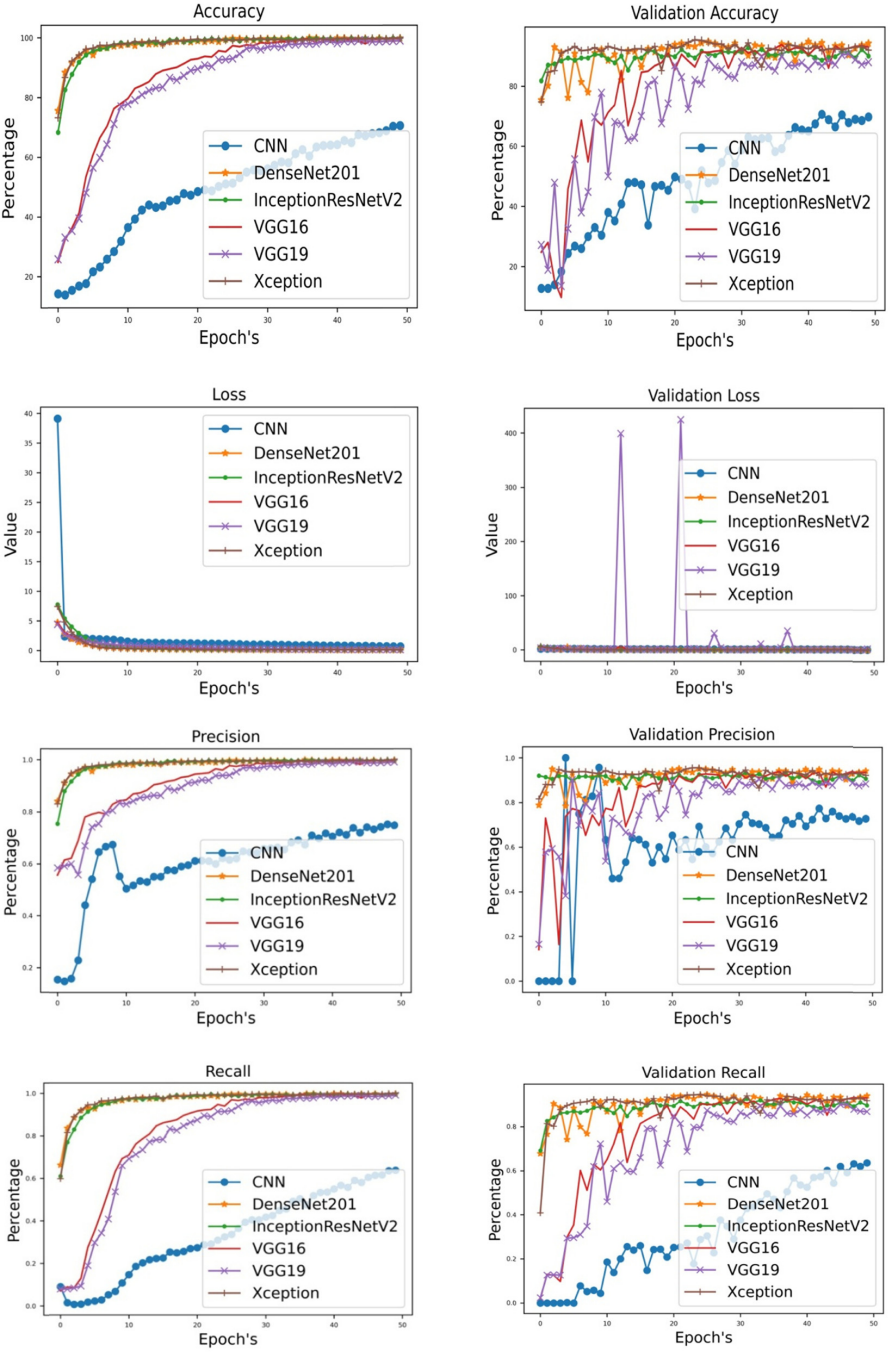
Plots for benchmark DNN.

As shown in Figure 4 and per the results of Table 2, Normal Result DNN showed that the accuracy of the model was approximately 70.53%, indicating that around 70.53% of the survival outcomes in the normal dataset was correctly predicted by the DNN. The precision value of 75.12% suggested that the DNN had a relatively high ability to correctly identify positive cases within the normal dataset. The recall value of 63.73% indicated that the model captured about 63.73% of the actual positive cases present in the normal dataset. The loss value of 0.696 reflected the discrepancy between the predicted survival outcomes and the actual outcomes, with a lower value indicating better performance. Overall, the Normal Result DNN demonstrated moderate performance in predicting survival outcomes for the noncancerous (normal) tissue, with room for improvement in terms of accuracy and recall. Further analysis and fine-tuning of the model may be necessary to enhance its performance. Table 3 presents results of federated learning (FL).

**Table 3:** Results of FL

	Training/validation	Data	Accuracy	Loss	Precision	Recall
0	Training	IID	99.97	0.07	99.97	99.92
1	Training	Non-IID	100	0.08	100	99.95
0	Validation	IID	93.02	0.38	92.62	93.28
1	Validation	Non-IID	94.34	0.29	94.2	94.72

**Figure 5:**
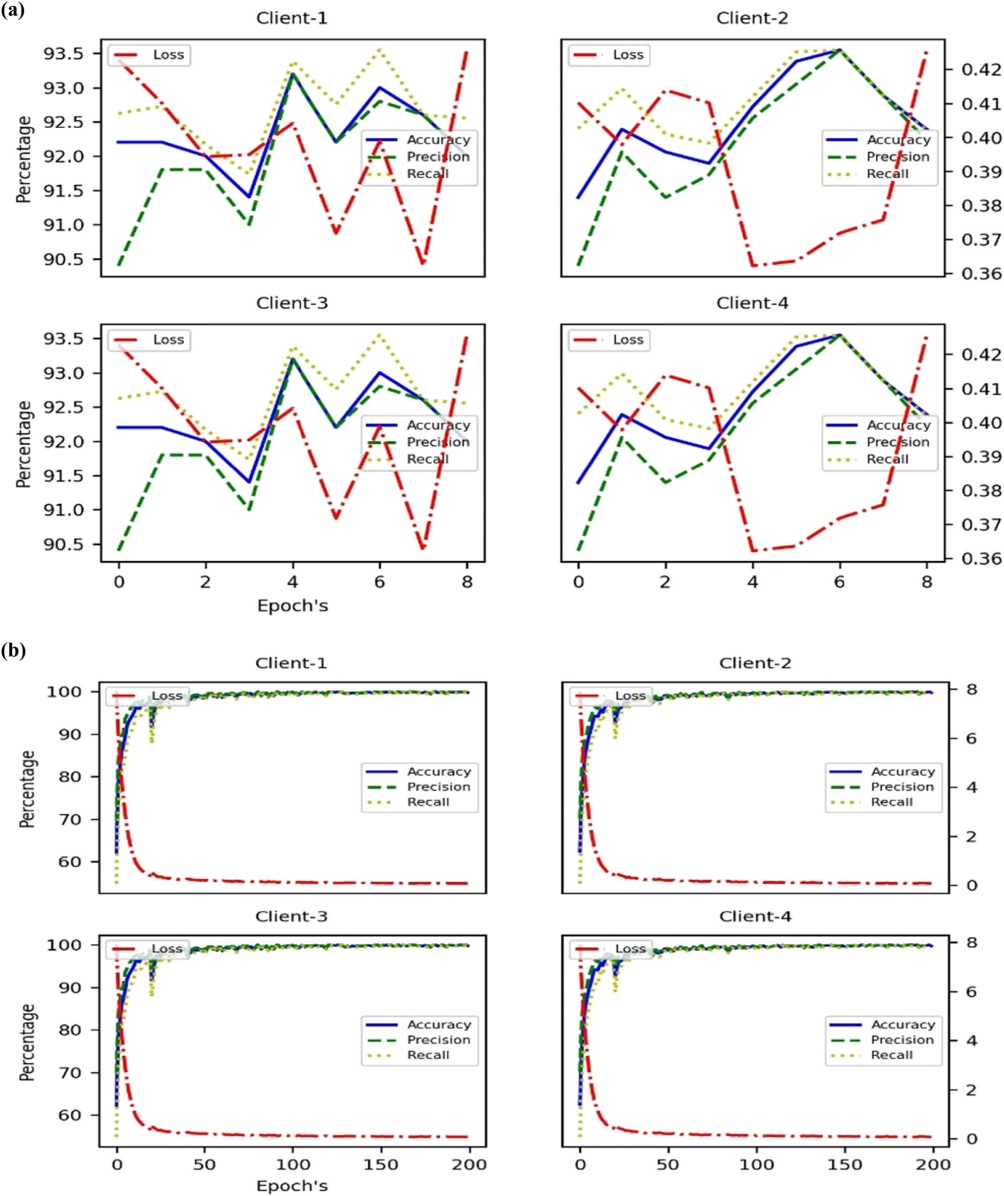
Plot for Server & Client IID Evaluation and IID training: (a) communication round wise server evaluation and (b) epoch wise client training.

The FL Results for Server & Client IID Evaluation and IID Training shown in Figure 5 and Table 3 data presents the performance metrics of the FL approach used in the research article. The results are divided into training and validation subsets, further categorized into IID (independent and identically distributed) and non-IID (non-independent and non-identically distributed) data. Here is a detailed analysis of the FL results. For training, IID data, FL model achieved an accuracy of 99.97% on the training data with IID distribution, indicating that it accurately predicted survival outcomes for the training samples. The loss value of 0.07 indicated a relatively low discrepancy between the predicted outcomes and the actual outcomes, implying good model performance. The precision value of 99.97% reflected the model’s ability to correctly identify positive cases within the training data. The recall value of 99.92% demonstrated the model’s capacity to capture most actual positive cases in the training data.

**Figure 6:**
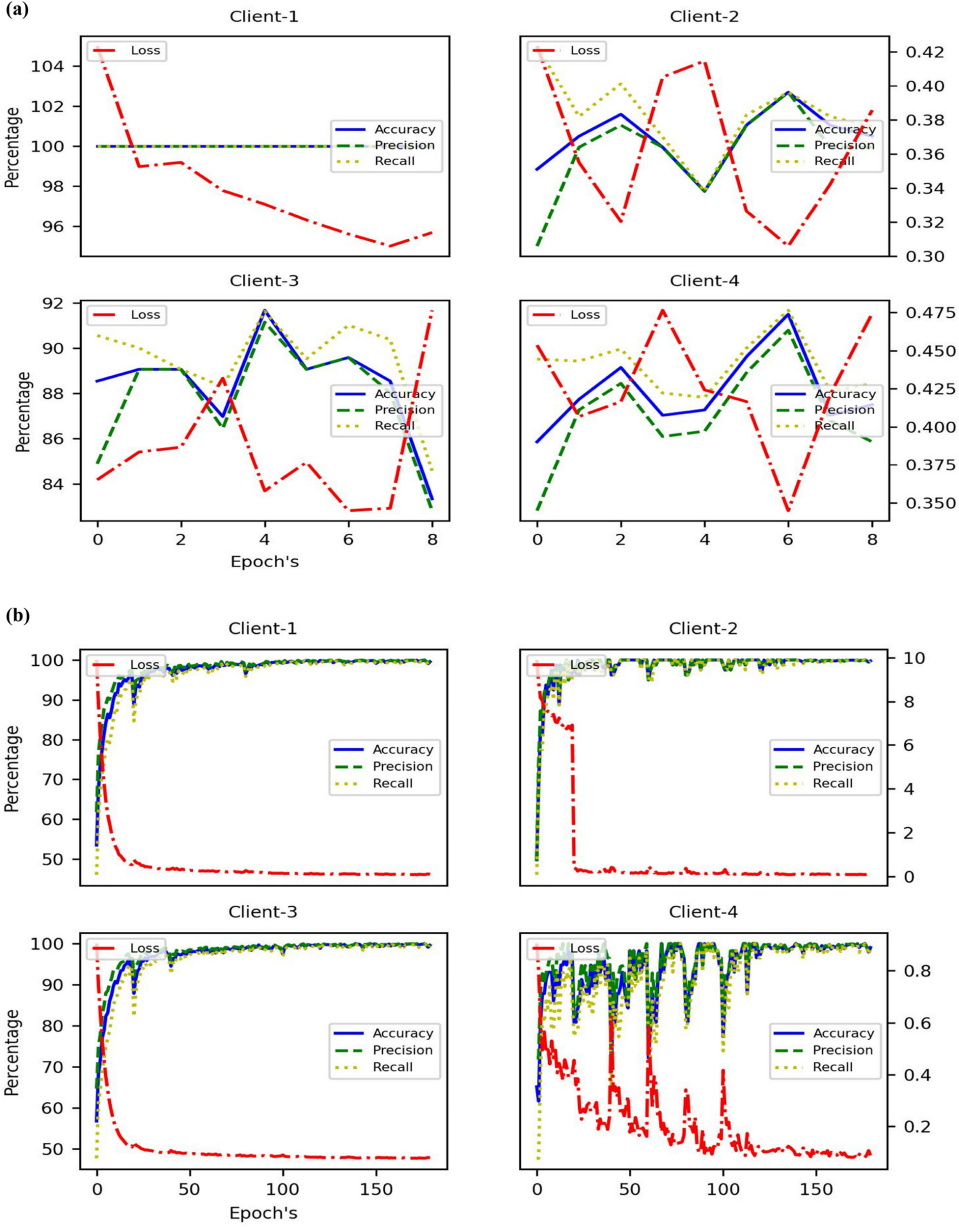
Plot for Server & Client NIID Evaluation and NIID training: (a) communication round wise server evaluation and (b) epoch wise client training.

In Figure 6, for non-IID data, the FL model achieved a perfect accuracy of 100% on the training data with non-IID distribution, indicating flawless predictions of survival outcomes for the training samples. The low loss value of 0.08 indicated a minimal discrepancy between predicted and actual outcomes in the training data. The model achieved a perfect precision of 100%, correctly identifying all positive cases within the training data with non-IID distribution. The recall value of 99.95% indicated the model’s high ability to capture a large proportion of actual positive cases in the training data. For validation of IID data, the FL model achieved an accuracy of 93.02% on the IID validation data, indicating reasonably accurate predictions of survival outcomes. The loss value of 0.38 indicated a moderate discrepancy between predicted and actual outcomes in the IID validation data. The precision value of 92.62% reflected the model’s ability to correctly identify positive cases within the IID validation data. The recall value of 93.28% demonstrated the model’s capacity to capture a significant proportion of actual positive cases in the IID validation data. For non-IID data, the FL model achieved an accuracy of 94.34% on the non-IID validation data, indicating good predictive performance on this subset. The loss value of 0.29 suggested a relatively low discrepancy between predicted and actual outcomes in the non-IID validation data. The precision value of 94.20% indicated the model’s ability to correctly identify positive cases within the non-IID validation data. The recall value of 94.72% demonstrated the model’s capacity to capture a substantial proportion of actual positive cases in the non-IID validation data.

The FL approach demonstrated strong performance in predicting survival outcomes in both the training and validation datasets. The model achieved high accuracy, low loss, and impressive precision and recall values across both IID and non-IID data subsets. These results indicate the effectiveness of the FL method in capturing patterns and making accurate predictions while accounting for the distributed and heterogeneous nature of the data.

## 5 Discussion

The research article compared and analyzed several deep learning architectures, including CNN, DenseNet201, InceptionResNetV2, VGG16, VGG19, and Xception, for their performance in prognostic survival prediction in human colorectal cancer histology. The accuracy metric measures the overall correctness of the models in predicting survival outcomes. DenseNet201, InceptionResNetV2, VGG16, and Xception achieved high accuracy values, ranging from 99.85 to 99.95%, indicating their strong predictive capabilities. CNN and VGG19 obtained slightly lower accuracy values but still demonstrated respectable performance. Precision measures the proportion of correctly identified positive cases, while recall evaluates the ability to capture all positive cases. DenseNet201, InceptionResNetV2, VGG16, and Xception consistently achieved high precision and recall values, suggesting their effectiveness in identifying and capturing positive cases. CNN and VGG19 also demonstrated reasonable precision and recall values, although slightly lower than the other architectures. The loss metric quantifies the discrepancy between predicted and actual survival outcomes. DenseNet201, InceptionResNetV2, and Xception exhibited lower loss values, indicating better alignment between predicted and actual outcomes. CNN, VGG16, and VGG19 achieved relatively higher loss values, suggesting a larger discrepancy. When considering validation accuracy, precision, recall, and loss, DenseNet201 and Xception consistently performed well across the different architectures. These models achieved high validation accuracy, precision, and recall, indicating their ability to generalize well to unseen data. VGG16 and InceptionResNetV2 also exhibited respectable performance on the validation set, while CNN and VGG19 showed comparatively lower validation metrics. DenseNet201, InceptionResNetV2, VGG16, and Xception stood out as the top-performing architectures in terms of accuracy, precision, recall, and validation metrics. These models demonstrated strong predictive capabilities and generalization to unseen data. CNN and VGG19, while achieving relatively lower metrics, still showed reasonable performance. The comparison and analysis of these architectures provide insights into their suitability for prognostic survival prediction in CRC histology and help researchers select the most effective models for similar tasks.

The proposed framework of the research article addresses significant deficiencies in the current body of literature by undertaking a thorough comparative analysis of diverse deep learning architectures, namely, CNN, DenseNet201, InceptionResNetV2, VGG16, VGG19, and Xception, with the aim of prognostic survival prediction in CRC histology. This study aims to fill a notable void in the existing literature, as prior research must frequently incorporate comprehensive and methodical architectural comparisons. The system effectively integrates training and validation measures, improving the evaluation of real-world applicability, a factor commonly neglected in clinical prediction tasks. Moreover, the research publication enhances transparency and reproducibility by providing comprehensive details regarding architecture, hyperparameters, and metrics. This contribution facilitates the replication and further development of this study into the broader research community. Compared to existing methodologies, the framework distinguishes itself by its comprehensive examination of architectural possibilities, emphasis on validation and generalization, and multifaceted evaluation approach, which collectively offer a thorough assessment of model performance.

## 6 Conclusion

The research has made several significant contributions to the field of prognostic survival prediction and deep learning in healthcare. The research developed and evaluated multiple deep learning models, including CNN, DenseNet201, InceptionResNetV2, VGG16, VGG19, and Xception, for prognostic survival prediction in CRC histology. These models demonstrated high accuracy, precision, recall, and other performance metrics, showcasing their potential for accurate survival prediction. The research employed visualization and interpretation techniques to enhance the understanding of deep learning models. Through activation maps, feature visualization, and attention mechanisms, the researchers visualized and interpreted the models’ decision-making process, providing insights into the important features and regions that influence survival predictions. The research utilized a large dataset of histopathological images of CRC and healthy tissue, providing a valuable resource for further studies in the field. In addition, the researchers implemented preprocessing techniques such as normalization and augmentation to optimize the performance of the deep learning models. The research findings have practical applications and clinical significance in the field of prognostic survival prediction in CRC histology. The developed deep learning models can aid in personalized medicine by accurately predicting survival outcomes for individual CRC patients. This information can assist clinicians in tailoring treatment plans and interventions based on the patient’s specific prognosis, optimizing patient care and outcomes. The models can serve as decision support tools for clinicians, providing additional insights and guidance in the interpretation of histopathological images. By leveraging the models’ predictions and visualizations, clinicians can make more informed decisions about treatment strategies, follow-up protocols, and patient management. The research findings can contribute to the identification and validation of biomarkers associated with CRC prognosis. The visualization and interpretation techniques help reveal the relevant features and patterns in histopathological images that have a significant impact on survival predictions. This knowledge can guide further research into biomarker discovery and facilitate the development of targeted therapies. The research provides a foundation for future studies in the field of prognostic survival prediction in CRC histology. The developed models, dataset, and preprocessing techniques can be utilized by researchers to expand upon the existing work, investigate new research questions, and develop more advanced models and methodologies. The research article has made important contributions to the field of prognostic survival prediction in CRC histology. The developed deep learning models, visualization techniques, and dataset provide valuable insights into survival prediction and have significant implications for personalized medicine, clinical decision-making, biomarker discovery, and future research. With further validation, refinement, and integration into clinical practice, the findings from this research can have a profound impact on improving patient outcomes in CRC and pave the way for advancements in prognostic prediction using deep learning in other areas of healthcare. Exploring model deployment in actual clinical settings and evaluating its influence on patient outcomes are crucial areas for future academic investigation.
